# Sarcomatoid hepatocellular carcinoma: A case report and review of the literature

**DOI:** 10.1097/MD.0000000000037641

**Published:** 2024-03-29

**Authors:** Chengyin Hu, Mingwei Zhao, Qiang Wei, Zhuo Chen, Baolei Zhao

**Affiliations:** a Department of Hepatobiliary Surgery, Binzhou Medical University Hospital, Binzhou, Shandong Province, China; b Department of Hepatobiliary Surgery, Binzhou Medical University Hospital, Binzhou, Shandong Province, China.

**Keywords:** case report, sarcomatoid hepatocellular carcinoma (SHC)

## Abstract

**Rationale:**

Sarcomatoid hepatocellular carcinoma (SHC) is a rare malignant tumor composed of both carcinoma and sarcoma components. It has atypical clinical symptoms and a high degree of malignancy, with rapid progression and a poor prognosis.

**Patient concerns:**

A 63-year-old female patient was admitted to our hospital with a chief complaint of fatigue present for more than 1 month and fever for 10 days.

**Diagnoses:**

This patient underwent an upper abdominal MRI plain scan and enhanced scan showed a solid tumor in the right lobe of the liver, with a size of approximately 4.7 cm × 4.0 cm × 6.5 cm, present as low signal on T1WI, slightly high signal on T2WI, and heterogeneous high signal on DWI. Multi-phase dynamic contrast-enhanced MR scan showed significant enhancement in the arterial phase and low enhancement in the portal and delayed phases. The pathology showed the tumor cells to be positive for cytokeratin (CK), Vimentin, EMA, CD34, cyclinD1, negative for CK8, CK19, CK20, SMA, Desmin, S-100, CD117, Dog-1, Hepar-1, SOX-10 and ALK, and Ki-67 approximately 50%, which confirmed the diagnosis of SHC.

**Interventions:**

Laparoscopic right posterior lobe of liver resection was conducted, and the postoperative pathology revealed the presence of SHC.

**Outcomes:**

The patient was discharged 9 days after the surgery without any complications. There has been no evidence of recurrence at the 1 month, however bilateral pleural metastases appeared during the follow-up 3 months after surgery.

**Lessons:**

SHC is a rare and aggressive liver cancer. So far, there is still a lack of effective therapeutic strategy, and the prognosis was dismal even though patients received radical surgical resection.

## 1. Introduction

Sarcomatoid carcinoma is a rare malignant tumor that exhibits both epithelial and mesenchymal characteristics and can occur in various organs throughout the body, the lung was the most common primary tumor site, followed by uterus, breast, and alimentary canal.^[1–4]^ Sarcomatoid hepatocellular carcinoma (SHC) is an uncommon malignant liver tumor with few cases recorded worldwide, accounting for 1.8% of all surgically resected cases of primary hepatic malignant. The reported prevalence of SHC in the literature ranges from 3.9% to 9.4% in autopsy cases.^[5,6]^ Herein, we report the case of a female patient suffering SHC who underwent surgical treatment. Aimed to analyze the clinical features, diagnosis, and treatment of SHC and combine with literature review.

## 2. Case presentation

### 2.1. Patient information

The patient is a 63-year-old female who was admitted to our hospital with a chief complaint of fatigue present for more than 1 month and a fever for 10 days. Approximately 1 month prior, the patient experienced fatigue syndrome without any obvious cause, accompanied by decreased appetite, but without abdominal pain, bloating, nausea, vomiting, or other discomfort. However, 10 days before admission, the patient experienced recurrent high fever. Physical examination upon admission revealed percussion pain in hepatic region, and no other positive signs were found.

### 2.2. Laboratory examination and imaging findings

**Figure 1.**
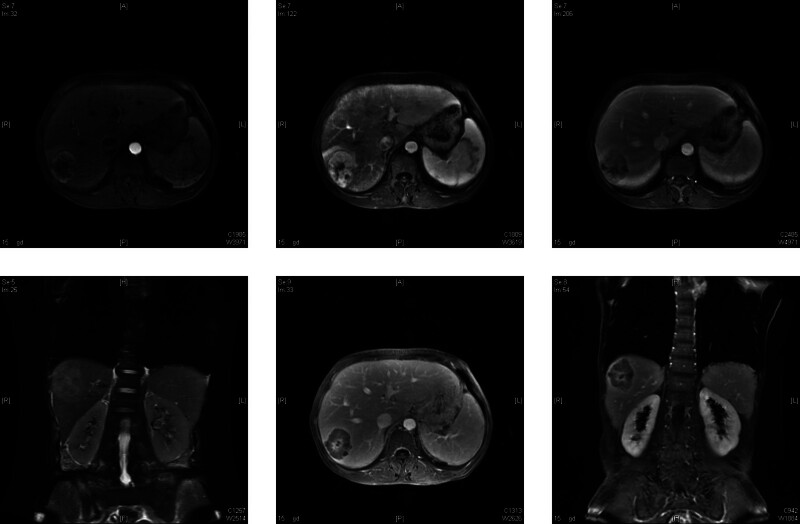
An abnormal mass was identified in the right lobe of the liver during an abdominal MRI scan with plain and dynamic enhancement.

Laboratory tests indicated elevated white blood cells (17.3 × 10^9^/L) and neutrophil percentage (86.1%), accompanied with a significant drop in blood hemoglobin (78 g/L), and increase of platelet count (463 × 10^9^/L). Blood biochemical examination prompts that the level of alanine aminotransferase, aspartate aminotransferase, alkaline phosphatase, γ-glutamyl transferase, total bilirubin within the normal range and a significant drop of albumin (22.90 g/L). The serum levels of CA125, CEA, CA19-9, and alpha-fetoprotein (AFP) were also measured, and no obvious abnormalities were found. Following admission, the patient underwent an upper abdominal MR plain scan and enhanced scan which showed a solid tumor in the right lobe of the liver, with a size of approximately 4.7 cm × 4.0 cm × 6.5 cm, present as low signal on T1WI, slightly high signal on T2WI, and heterogeneous high signal on DWI. Multi-phase dynamic contrast-enhanced MR scan showed significant enhancement in the arterial phase and low enhancement in the portal and delayed phases (Fig. 1).

### 2.3. Therapeutic interventions and histopathological findings

**Figure 2.**
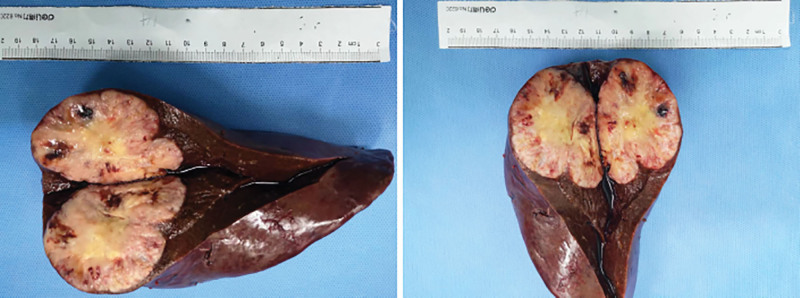
Completely resected surgical specimen.

**Figure 3.**
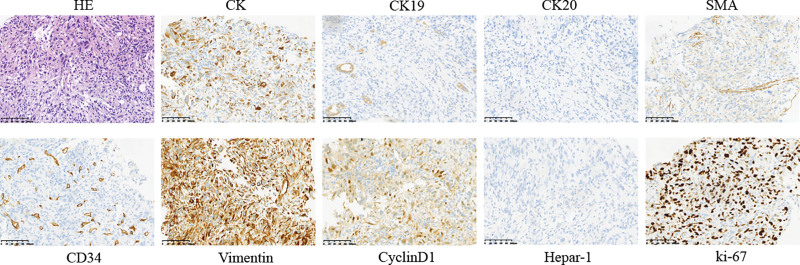
Histopathological and immunohistochemical analysis of ultrasound-guided tumor biopsy.

To further clarify the histological types of hepatic tumor, ultrasound-guided liver biopsies were performed and the pathology showed the tumor cells to be positive for cytokeratin (CK), Vimentin, EMA, CD34, cyclinD1, negative for CK8, CK19, CK20, SMA, Desmin, S-100, CD117, Dog-1, Hepar-1, SOX-10 and ALK, and Ki-67 approximately 50% (Fig. 2), which confirmed the diagnosis of SHC. Subsequently, we performed laparoscopic right posterior lobe of liver resection. During the surgery, a solid tumor measuring 7 cm × 6 cm × 5 cm was identified in the posterior lobe of the liver, no metastasis was found in the liver and other organs in the abdominal cavity. The tumor was completely removed (Fig. 3). The postoperative pathological report revealed a malignant tumor in the liver tissue, consistent with SHC based on immunohistochemistry results. Immunohistochemical evaluation showed partial positivity for CK, positive staining for Vimentin, and scattered weak positivity for CK19. Negative staining was observed for CK20, HepPar-1, AFP, and glypican-3. The Ki-67 proliferation rate was estimated to be around 70%. The preoperative and postoperative pathological findings were in agreement.

### 2.4. Follow-up and outcomes

**Figure 4.**
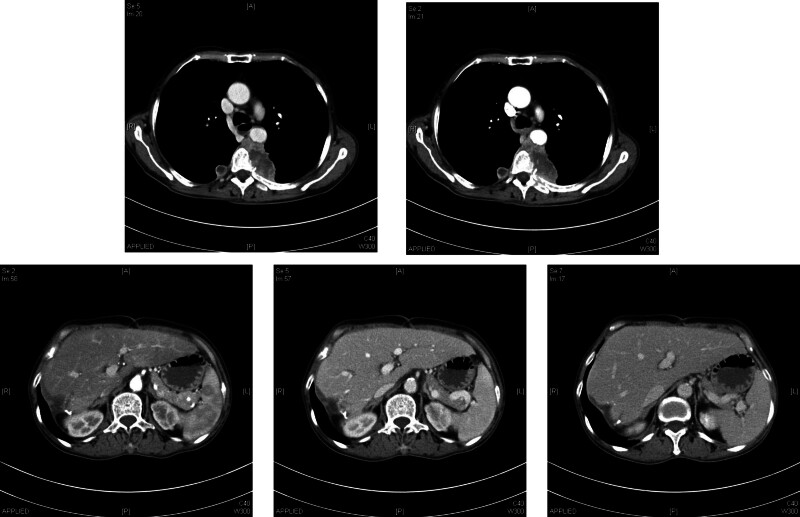
Chest and abdominal CT examination at 3 months postsurgery.

The patient recovered well after surgery. One week after surgery, a blood routine examination showed that white blood cells, hemoglobin, and platelets had returned to normal range and the patient was discharged 9 days after surgery without any complications. There has been no evidence of recurrence at the 1-month follow-up. However, at a follow-up of 3 months after surgery, CT examination showed bilateral pleural metastasis, but there was no recurrence or metastasis in the liver (Fig. 4).

## 3. Discussion

SHC is a neoplasm that contains both carcinoma and sarcomatoid components. It can be classified as SHC, intrahepatic sarcomatoid cholangiocarcinoma, and undifferentiated carcinoma based on the cellular composition of the carcinoma.^[7,8]^ It is important to note that there are significant differences between hepatocellular carcinoma (HCC) and SHC. The latter displays a more invasive tumor biology profile, a higher propensity for metastasis, and lower resectability, with an increased risk of postoperative recurrence.^[9,10]^ Nonsurgical therapeutic modalities, such as transarterial chemoembolization, radiofrequency ablation, and percutaneous ethanol injection, may cause damage and degeneration of hepatic cells. However, this can promote the transition of HCC into sarcomatoid phenotype.^[11–13]^ Rare cases have been reported in the literature of hepatocellular carcinoma or intrahepatic cholangiocarcinoma transforming into a sarcoma-like morphology, even in cases where no recurrent intervention therapy had been received.

SHC typically presents with atypical symptoms, including digestive system disturbances such as abdominal distension and pain, tumor-related weight loss, and constitutional symptoms like low-grade fever and jaundice. The nonspecific nature of these presentations poses challenges in diagnosing SHC. Literature reports suggest that some patients may exhibit hyperthermia and leukocyte reaction-like conditions. Early detection and diagnosis of SHC require a comprehensive consideration of key points and further clinical evaluation.

Laboratory tests lack specificity and are often characterized by a decrease in hemoglobin, an increase in white blood cell count and C-reactive protein. According to literature reports, the levels of AFP, CA199, and CEA are often not elevated. Preoperative ultrasound, CT, and magnetic resonance imaging (MRI) examinations are helpful for the diagnosis of SHC. Ultrasound examination shows that the tumor is a hypoechoic lesion with uneven echogenic changes inside, with or without capsule. CT examination shows marginal enhancement of tumors in the arterial and portal phases, with no or uneven enhancement visible in the central area, which is significantly different from the CT findings of HCC. MRI shows weak signal intensity on T1WI MRI, but high signal intensity on T2WI. Dynamic contrast-enhanced MR imaging shows that hyper-enhancement during the arterial phase and washout in the portal and delayed phases, while the central scar area shows delayed enhancement. The MRI findings are consistent with previous literature reports. The SHC presented intense uptake of 18F-FDG, as a result, PET-CT has great significance in the diagnosis and evaluation of postoperative recurrence for SHC.

Histopathological and immunohistochemical analysis is the main method for diagnosis of SHC. The pathological examination of SHC tissue shows that it contains both malignant epithelial cells and sarcoma-like components, and the proportion of sarcoma-like components is mostly more than 50%, scanning electron microscopy examination can reveal the migration status of 2 types of cells. Immunohistochemical examination showed positive expression both of epithelial cell marker CK and sarcoma-like component marker Vimentin.

To date, there is still a lack of effective treatment options for patients diagnosed with SHC. Surgical resection remains the primary treatment option. However, due to the aggressive nature of SHC and its tendency to recur, surgical treatment does not bring satisfactory treatment outcomes. There are also other treatment measures applied to SHC, including liver transplantation, radiation therapy, and chemotherapy. Liver transplantation may be a viable therapeutic option in certain cases. However, its feasibility is limited by donor scarcity and the rarity of SHC. Radiotherapy^[14]^ and chemotherapy^[15]^ are commonly used to treat SHC. However, the effectiveness of these treatments is limited due to the inherent resistance and the occurrence of adverse reactions.

We present a rare case of SHC. Currently, the diagnosis and treatment of SHC still face significant challenges. Future studies are urgently needed to explore more effective therapies to extend the survival period of SHC patients and improve their quality of life.

## Acknowledgments

We express our gratitude to all individuals who participated in this study, including patients and medical professionals responsible for disease diagnosis and treatment.

## Author contributions

**Conceptualization:** Chengyin Hu.

**Data curation:** Mingwei Zhao, Qiang Wei.

**Investigation:** Mingwei Zhao, Qiang Wei, Zhuo Chen.

**Project administration:** Baolei Zhao.

**Resources:** Mingwei Zhao, Zhuo Chen.

**Software:** Qiang Wei.

**Supervision:** Baolei Zhao.

**Writing – original draft:** Chengyin Hu.

**Writing – review & editing:** Baolei Zhao.
